# Differential Effects of Oral Antidiabetic Drugs on Skeletal Muscle Mass and Hemoglobin Levels in Adults with Type 2 Diabetes Mellitus: A Prospective Real-World Cohort Study

**DOI:** 10.3390/jcm15083172

**Published:** 2026-04-21

**Authors:** Fatma Pınar Ziyadanoğlu, Ece Çiftçi Öztürk, Gamze Şengün, Seher İrem Şahin, Büşra Çetintulum Aydın, Hayriye Esra Ataoğlu

**Affiliations:** Department of Internal Medicine, Haseki Training and Research Hospital, Istanbul 34093, Türkiye; eciftci3506@gmail.com (E.Ç.Ö.); gamzesengun@gmail.com (G.Ş.); siremcetin@gmail.com (S.İ.Ş.); busracetintulum@hotmail.com (B.Ç.A.); eataoglu@gmail.com (H.E.A.)

**Keywords:** type 2 diabetes mellitus, pioglitazone, SGLT2 inhibitors, skeletal muscle mass, hemoglobin, real-world study, body composition, erythropoiesis

## Abstract

**Background/Objectives:** Beyond glycemic control, oral antidiabetic drugs (OADs) may exert class-specific effects on muscle mass and hematologic parameters. However, real-world evidence comparing these effects across OAD classes remains limited. This study aimed to evaluate the differential effects of commonly prescribed OADs on skeletal muscle mass (SMM) and hemoglobin (Hb) levels in adults with type 2 diabetes mellitus (T2DM). **Methods:** In this prospective observational cohort study, 60 adults with newly initiated OAD therapy were followed for six months at a tertiary care center in Türkiye. Patients were classified according to the OAD class newly added to their regimen (metformin, sulfonylureas, dipeptidyl peptidase-4 inhibitors, pioglitazone, or sodium–glucose cotransporter-2 inhibitors [SGLT2-i]). Multi-frequency bioelectrical impedance analysis was used to evaluate body composition, and hematologic parameters including Hb were obtained at both time points. To account for potential confounders—including age, sex, BMI, baseline Hb, and eGFR—binary logistic regression analyses were performed. **Results:** Patients initiated on pioglitazone (n = 11) demonstrated a borderline within-group increase in SMM in unadjusted analysis (median delta +0.17 kg, IQR −0.55 to +0.50; *p* = 0.050); however, this association was attenuated and no longer statistically significant after multivariable adjustment (OR 2.16, 95% CI 0.60–7.83; *p* = 0.240). In contrast, SGLT2-i users (n = 28) showed a significant increase in Hb (median delta +0.10 g/dL, IQR −0.30 to +0.50; *p* = 0.022), which remained significant after adjustment (OR 4.22, 95% CI 1.32–13.44; *p* = 0.015). Other OAD classes were not associated with meaningful changes in SMM or Hb. **Conclusions:** In this real-world prospective cohort, pioglitazone showed a trend toward increased SMM in unadjusted analysis that did not reach significance after adjustment, suggesting a hypothesis-generating signal warranting further investigation. SGLT2 inhibitors were independently associated with increased Hb levels, though the observed median increment was modest in absolute terms. These findings highlight potentially clinically relevant, non-glycemic effects of OAD classes and may inform individualized treatment selection, particularly in patients at risk of sarcopenia or anemia. Adequately powered, prospective studies are needed to validate and extend these preliminary observations.

## 1. Introduction

Type 2 diabetes mellitus (T2DM) represents a major global public health challenge, currently affecting more than 500 million individuals worldwide, with projections indicating a substantial increase in prevalence in the coming decades [1]. When T2DM coexists with obesity, the risk of cardiovascular events, functional impairment, and early mortality is further compounded [2,3]. Although optimizing glycemic control remains central to diabetes care, growing evidence has drawn attention to non-glycemic physiologic outcomes—particularly alterations in body composition and hematologic indices—as important determinants of long-term patient prognosis [3,4,5].

Skeletal muscle mass plays a central role in insulin sensitivity, metabolic regulation, and physical performance. Sarcopenia—defined as the progressive loss of skeletal muscle mass, strength, and function—is increasingly recognized as a complication of T2DM, with a pooled prevalence of approximately 18% reported in a systematic review of 28 studies involving 16,800 diabetic patients, and with rates exceeding 30% in certain older populations depending on diagnostic criteria and setting [6,7]. The relationship between sarcopenia and insulin resistance is bidirectional: reduced muscle mass impairs glucose uptake and worsens glycemic control, while chronic hyperglycemia and associated inflammatory and oxidative stress pathways accelerate muscle protein catabolism [6,7]. Beyond metabolic consequences, sarcopenia in T2DM is independently associated with increased risk of falls, frailty, hospitalization, and mortality, underscoring its clinical relevance as a therapeutic target. Despite this, there is limited real-world evidence on how different oral antidiabetic drugs (OADs) influence skeletal muscle mass, and whether treatment selection may be optimized to simultaneously address glycemic and musculoskeletal outcomes.

In routine clinical practice, particularly in middle-income healthcare settings such as Türkiye, treatment decisions are often influenced by drug accessibility and reimbursement policies. Advanced injectable therapies, including glucagon-like peptide-1 receptor agonists, may be limited in availability [8]. Consequently, frequently prescribed OADs—including metformin, sulfonylureas, DPP-4 inhibitors, pioglitazone, and SGLT2 inhibitors—constitute the foundation of pharmacologic management in this context. Although their glucose-lowering effects are well established, their potential class-specific, non-glycemic effects on skeletal muscle mass and hematologic parameters, including hemoglobin (Hb) and hematocrit (Hct), are less clearly defined [9,10,11].

Among these agents, pioglitazone, a thiazolidinedione acting through peroxisome proliferator-activated receptor gamma (PPAR-γ) activation, has been proposed to exert beneficial effects on muscle metabolism and preservation [10]. PPAR-γ activation influences skeletal muscle mitochondrial biogenesis, reduces intramyocellular lipid accumulation, and enhances oxidative phosphorylation capacity—mechanisms that may collectively support lean tissue preservation or accretion [12,13]. While pioglitazone acts predominantly on metabolic pathways within skeletal muscle, SGLT2 inhibitors exert distinct hematologic effects through separate mechanisms: by reducing plasma glucose via renal glycosuria and inducing natriuresis, they have been associated with stimulation of erythropoiesis through renal hypoxia-inducible factor (HIF) signaling and suppression of hepcidin, potentially leading to sustained increases in Hb and Hct levels [9,10,11]. These mechanistic differences suggest that individual OAD classes may carry distinct physiologic footprints beyond their shared glucose-lowering activity. However, prospective, real-world comparative data simultaneously evaluating these class-specific effects on body composition and hematologic parameters across multiple OAD classes remain limited.

Body composition assessment in clinical settings is commonly performed using bioelectrical impedance analysis (BIA), a practical and cost-effective method. Although dual-energy X-ray absorptiometry (DEXA) is considered the reference standard, validated BIA devices such as TANITA MC-780MA provide reproducible and accessible measurements of lean mass and fat distribution in routine care [13,14].

In this context, the present study was designed as a prospective, real-world evaluation of commonly prescribed OAD classes in a tertiary care setting. The primary objective was to investigate their differential effects on skeletal muscle mass and hematologic parameters over a six-month follow-up period. We hypothesized that pioglitazone would be associated with preservation or increase in skeletal muscle mass, whereas SGLT2 inhibitors would be associated with increased hemoglobin levels.

## 2. Materials and Methods

### 2.1. Study Design and Setting

This prospective observational cohort study was conducted at Haseki Training and Research Hospital, a tertiary care center in Türkiye, between June 2021 and February 2022. The study was designed to reflect real-world prescribing practices in routine clinical care. Oral antidiabetic therapy was initiated at the discretion of treating physicians based on patients’ glycemic status, comorbidities, renal function, body weight, and prior treatment history. No protocol-driven allocation or randomization was performed.

Drug dosing followed standard clinical practice. Pioglitazone was prescribed at the commonly used dose of 30 mg/day, reflecting national availability, while SGLT2 inhibitors (empagliflozin or dapagliflozin) were administered at standard daily doses of 10 mg. The study was designed to generate hypothesis-forming real-world evidence rather than establish causal relationships.

### 2.2. Participants

Eligibility criteria included age 18–75 years, a verified T2DM diagnosis, and initiation of a new OAD regimen (metformin, sulfonylureas, DPP-4 inhibitors, pioglitazone, or SGLT2 inhibitors) within the preceding two weeks. Patients were excluded if they had type 1 diabetes mellitus, active pregnancy or lactation, concurrent insulin use, impaired renal function (eGFR below 60 mL/min/1.73 m^2^), hepatic insufficiency, significant neuromuscular or musculoskeletal pathology, hypoalbuminemia, a recent hospitalization, or a prior diagnosis of heart failure.

All participants provided written informed consent prior to enrollment. The study protocol was approved by the Ethics Committee of Haseki Training and Research Hospital (Approval No: 55-2021; 23 June 2021). A total of 60 consecutive eligible patients were included, and all completed the six-month follow-up.

### 2.3. Exposure Definition and Treatment Classification

Patients were categorized according to the OAD class newly initiated at baseline. Given the real-world nature of the study, combination therapy was permitted, and patients could be included in more than one exposure group. For class-specific analyses, two primary comparisons were defined: (i) pioglitazone users versus non-users, and (ii) SGLT2 inhibitor users versus non-users. This approach enabled evaluation of class-specific effects while preserving real-world prescribing patterns. In accordance with national reimbursement criteria in Türkiye at the time of the study, SGLT2 inhibitors were prescribed exclusively in combination with metformin; therefore, all SGLT2-i users in this cohort received concomitant metformin therapy.

### 2.4. Variables and Measurements

The primary outcomes were changes in skeletal muscle mass (SMM, kg) and hemoglobin (Hb, g/dL) over six months. Secondary variables included body mass index (BMI), body weight, extracellular fluid (ECF), hematocrit (Hct), and glycated hemoglobin (HbA1c). Potential confounders included age, sex, baseline BMI, baseline Hb, and estimated glomerular filtration rate (eGFR).

Patient sociodemographic and clinical information was collected via standardized face-to-face interviews and review of electronic medical records. Physical activity levels were categorized in line with the criteria outlined in World Health Organization guidelines.

Anthropometric measurements were obtained with calibrated equipment; BMI was derived as weight in kilograms divided by the square of height in meters. Body composition analysis was conducted using a validated multi-frequency bioimpedance device (TANITA MC-780MA, Tanita Corp., Tokyo, Japan). The device was calibrated according to the manufacturer’s standard operating procedures before each measurement session, using the built-in calibration protocol verified against reference weights. To standardize conditions across assessments, participants were instructed to observe an overnight fast, abstain from alcohol and caffeine for the preceding 24 h, and avoid vigorous exercise before testing.

Fasting venous blood samples were collected at baseline and six months. Laboratory analyses, including Hb, HbA1c, hematocrit (Hct), fasting blood glucose, albumin, lipid parameters, renal function tests (creatinine, urea, eGFR calculated using the CKD-EPI equation), and liver enzymes, were performed using standardized automated methods at the hospital’s accredited central laboratory. T2DM diagnosis was established according to American Diabetes Association criteria, defined as fasting plasma glucose ≥ 126 mg/dL, two-hour plasma glucose ≥ 200 mg/dL during a 75 g oral glucose tolerance test, HbA1c ≥ 6.5%, or a random plasma glucose ≥ 200 mg/dL in a symptomatic patient. Glycemic targets were individualized according to ADA recommendations based on patient age, comorbidity burden, and hypoglycemia risk. The use of artificial intelligence tools for text editing purposes during manuscript preparation was limited to grammar and language refinement; no AI tools were used for data generation, analysis, or scientific interpretation.

### 2.5. Bias and Study Size

To minimize selection bias, consecutive eligible patients were enrolled without prior knowledge of their assigned treatment group. Measurement bias was reduced through standardized protocols applied uniformly across all participants and time points. Given the observational design, residual confounding was addressed using multivariable adjustment. The prospective observational design was chosen deliberately to reflect real-world prescribing behavior and to capture physiologic changes occurring under routine clinical conditions, as opposed to the highly controlled and often selected populations of randomized controlled trials. The six-month follow-up duration was selected based on the expected timescale of measurable body composition changes detectable by bioimpedance analysis and early hematologic adaptations to OAD therapy in clinical practice. As all 60 enrolled patients completed the six-month follow-up without attrition, no imputation was required; the analysis is therefore equivalent to a per-protocol analysis. Medication adherence was not formally assessed through pill counts, pharmacy records, or diaries; continuity of treatment was inferred from prescription records and clinical documentation at each visit. This study was designed as a pilot, hypothesis-generating investigation; therefore, no formal sample size calculation was performed. Future adequately powered studies should incorporate formal power calculations based on the effect sizes observed herein.

### 2.6. Statistical Analysis

All statistical computations were conducted with IBM SPSS Statistics (version 26.0; IBM Corp., Armonk, NY, USA). Normality of distribution was examined using the Shapiro–Wilk test. Given the predominantly non-normal distribution of the study variables, within-group changes over the observation period were analyzed with the Wilcoxon signed-rank test, and intergroup differences were assessed using the Mann–Whitney U test.

To evaluate independent associations between drug exposure and outcomes, binary logistic regression models were constructed. Dependent variables were defined as increase versus no increase in SMM and Hb. Covariates included age, sex, baseline BMI, baseline Hb, and eGFR, entered simultaneously into the model (forced entry method; no stepwise selection was performed). Results of the regression models are presented as adjusted odds ratios (ORs) with corresponding 95% confidence intervals (CIs). Statistical significance was defined as a two-tailed *p*-value below 0.05.

## 3. Results

### 3.1. Participant Characteristics

A total of 60 patients were enrolled, and all completed the six-month follow-up without attrition. Patients were categorized according to the OAD initiated at baseline. Due to real-world prescribing patterns, most patients received combination therapy; therefore, treatment groups reflect drug initiation rather than exclusive use.

Baseline demographic and clinical characteristics are summarized in Table 1. The cohort comprised 34 women (56.7%) and 26 men (43.3%). No missing data were observed at baseline or follow-up. Participants completed a mean observation period of 6.0 ± 0.2 months. By BMI category, 6 patients (10.0%) had normal weight (BMI 18.5–24.9 kg/m^2^), 20 (33.3%) had overweight (BMI 25.0–29.9 kg/m^2^), and 34 (56.7%) had obesity (BMI ≥ 30 kg/m^2^), reflecting the metabolic profile characteristic of a real-world endocrinology outpatient cohort.

**Figure 1:**
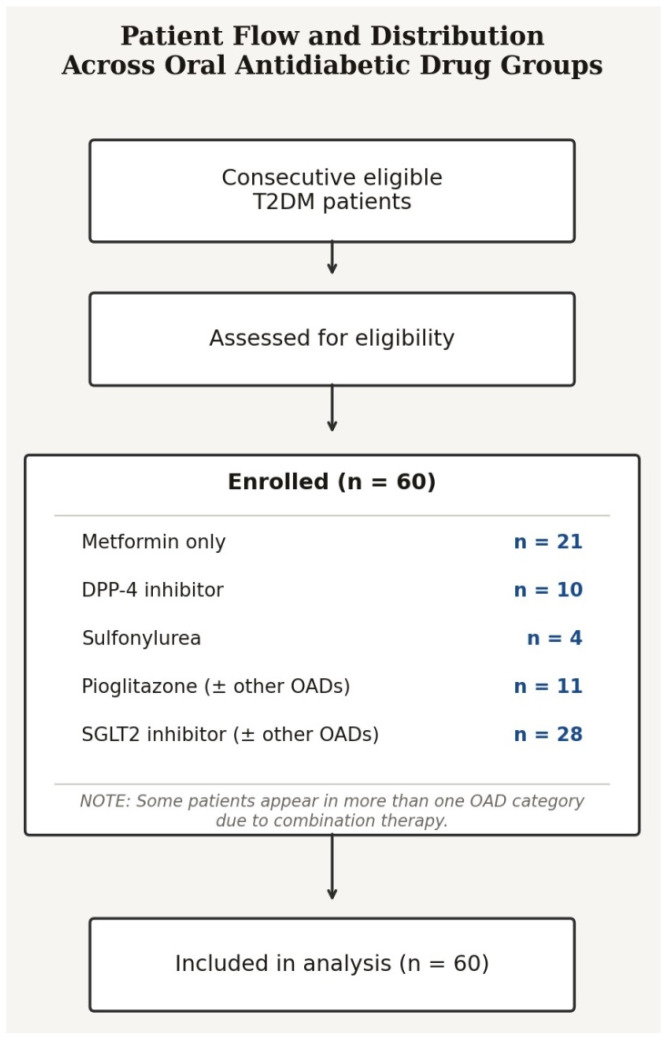
Patient flow and distribution across oral antidiabetic drug (OAD) treatment groups. Sixty consecutively enrolled adults with T2DM were included. Since each patient was classified according to the OAD newly added at baseline and polypharmacy was permitted, individual patients may be represented in multiple treatment categories. Metformin only: n = 21; DPP-4 inhibitor: n = 10; sulfonylurea: n = 4; pioglitazone (± other OADs): n = 11; SGLT2 inhibitor (± other OADs): n = 28. DPP-4: dipeptidyl peptidase-4; SGLT2-i: sodium–glucose cotransporter-2 inhibitor; OAD: oral antidiabetic drug.

The distribution of patients across OAD treatment groups is shown in Figure 1. Regarding treatment complexity, 36 patients (60.0%) received one newly initiated OAD, 16 (26.7%) received two, and 8 (13.3%) received three OAD agents simultaneously. Among SGLT2-i users (n = 28), concomitant newly initiated agents included metformin in 10 (35.7%), DPP-4 inhibitors in 7 (25.0%), and sulfonylureas in 3 (10.7%). Among pioglitazone users (n = 11), co-initiated agents included DPP-4 inhibitors in 5 (45.5%), metformin in 3 (27.3%), and sulfonylureas in 2 (18.2%). As previously noted, all SGLT2-i users received concomitant metformin in accordance with national reimbursement criteria, whether initiated simultaneously or already in use at baseline.

### 3.2. Overall Changes in the Cohort

Within the overall cohort, significant improvements were observed in several parameters over the six-month follow-up period. Reductions in body weight, BMI, and HbA1c, and fasting blood glucose were noted, consistent with effective glycemic management across OAD classes. The overall change in hemoglobin was not statistically significant (*p* = 0.659), nor was the change in extracellular fluid (*p* = 0.055), indicating that aggregate hematologic and fluid changes at the whole-cohort level were largely driven by group-specific effects rather than a uniform response (Table 2).

### 3.3. Pioglitazone Group

A total of 11 patients were newly initiated on pioglitazone. Over six months, these patients demonstrated a borderline increase in SMM compared with non-users. The median change in SMM was +0.17 kg (IQR: −0.55 to +0.50) in the pioglitazone group versus −0.36 kg (IQR: −1.04 to +0.18) in non-users (*p* = 0.050).

No statistically significant change in hemoglobin was observed in the pioglitazone group (*p* = 0.482). Notably, extracellular fluid (ECF) did not increase significantly in the pioglitazone group compared with non-users (+0.15 kg vs. −0.13 kg; *p* = 0.095), and hematocrit delta did not differ between groups (*p* = 0.580). These findings suggest that the observed increase in SMM is unlikely to be solely attributable to fluid retention, and may reflect a genuine change in lean tissue (Table 3).

### 3.4. SGLT2 Inhibitor Group

A total of 28 patients were newly initiated on SGLT2 inhibitors. These patients demonstrated a significant increase in hemoglobin over six months. The median change in Hb was +0.10 g/dL (IQR: −0.30 to +0.50) in SGLT2 inhibitor users versus −0.10 g/dL (IQR: −0.60 to +0.30) in non-users (*p* = 0.022).

No statistically significant change in SMM was observed in this group (*p* = 0.386). Importantly, hematocrit delta did not reach statistical significance between groups (+1.15% vs. −0.10%; *p* = 0.088), and extracellular fluid changes were comparable between SGLT2-i users and non-users (*p* = 0.221). The absence of a significant reduction in ECF argues against hemoconcentration as the primary driver of hemoglobin elevation, pointing instead toward a true erythropoietic mechanism (Table 4).

### 3.5. Other OAD Groups

Patients treated with metformin, DPP-4 inhibitors, or sulfonylureas did not demonstrate statistically significant changes in SMM or hemoglobin during follow-up. User versus non-user analyses were formally conducted for each of these OAD classes using the same Mann–Whitney U test approach applied to the pioglitazone and SGLT2-i groups. No statistically significant between-group differences were identified: metformin users (n = 32) versus non-users showed no significant difference in SMM delta (*p* = 0.733) or Hb delta (*p* = 0.321); DPP-4 inhibitor users (n = 16) versus non-users showed *p* = 0.114 for SMM and *p* = 0.118 for Hb; and sulfonylurea users (n = 5) versus non-users showed *p* = 0.574 for SMM and *p* = 0.862 for Hb. Given the absence of clinically meaningful differences, no additional tables were generated for these groups.

### 3.6. Multivariable Analysis

**Figure 2:**
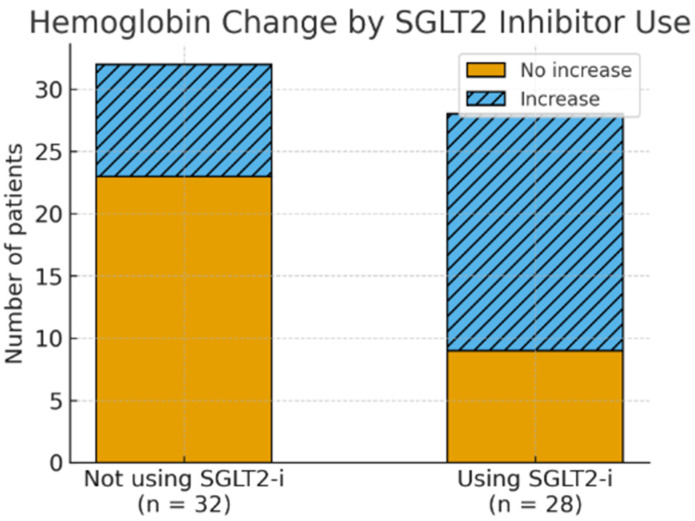
Distribution of hemoglobin changes among patients with type 2 diabetes mellitus according to sodium–glucose cotransporter-2 inhibitor (SGLT2-i) therapy status. Of patients receiving SGLT2-i (n = 28), 19 experienced a hemoglobin increase and 9 did not. Among non-users (n = 32), 9 experienced an increase and 23 did not. SGLT2-i use was significantly associated with hemoglobin increase (OR 4.22; 95% CI 1.32–13.44; *p* = 0.015).

Multivariate logistic regression analysis adjusted for age, sex, baseline BMI, baseline Hb, and eGFR demonstrated that SGLT2 inhibitor use was independently associated with an increase in hemoglobin (OR 4.22; 95% CI 1.32–13.44; *p* = 0.015) (Figure 2).

**Figure 3:**
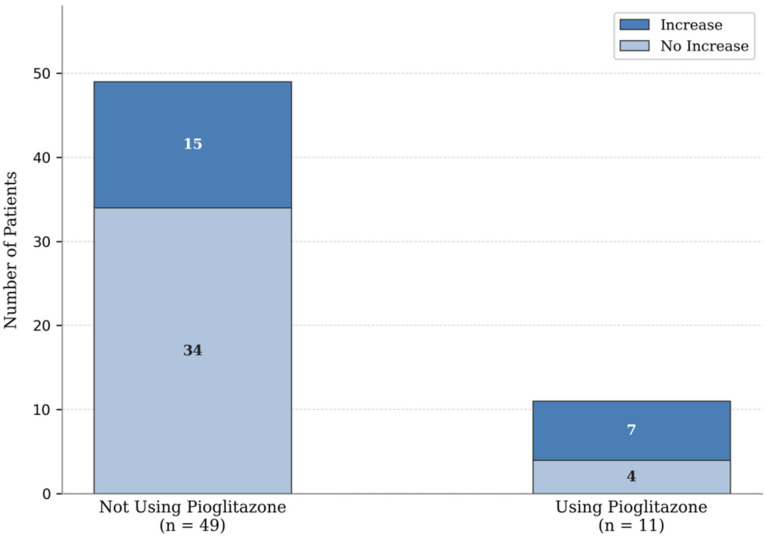
Distribution of skeletal muscle mass (SMM) changes according to pioglitazone therapy among patients with type 2 diabetes mellitus. Bars represent the number of patients showing an increase versus no increase in SMM over 6 months. Of the 11 pioglitazone-treated patients, 7 showed an SMM increase and 4 did not. Logistic regression indicated a non-significant trend toward SMM gain with pioglitazone use (OR 2.16; 95% CI 0.60–7.83; *p* = 0.240).

In contrast, pioglitazone use showed a positive but non-significant association with SMM increase (OR 2.16; 95% CI 0.60–7.83; *p* = 0.240) (Figure 3).

No significant associations were observed between changes in SMM or hemoglobin and age, sex, BMI, HbA1c, or physical activity.

### 3.7. Additional Notes

Because most patients received combination therapy, exposure groups reflect drug initiation rather than strict monotherapy. Values presented as ranges correspond to interquartile ranges (IQRs).

## 4. Discussion

In this prospective real-world cohort of adults with T2DM, two class-specific, non-glycemic patterns were observed over six months: pioglitazone initiation was associated with a trend toward increased skeletal muscle mass (SMM) in unadjusted analysis—a signal that was attenuated after adjustment and should be interpreted as hypothesis-generating—while SGLT2 inhibitor use was associated with a robust, independently confirmed increase in hemoglobin (Hb). These findings support the concept that oral antidiabetic drugs may exert differential physiologic effects beyond glycemic control, with potential implications for individualized treatment strategies.

### 4.1. Pioglitazone and Skeletal Muscle Mass

As a thiazolidinedione, pioglitazone enhances peripheral insulin sensitivity by acting as a selective agonist of peroxisome proliferator-activated receptor gamma (PPAR-γ), thereby modulating metabolic processes within skeletal muscle [10]. In the present study, patients initiated on pioglitazone demonstrated an increase in SMM in unadjusted analyses, consistent with mechanistic evidence suggesting improvements in mitochondrial function, reduced intramyocellular lipid accumulation, and enhanced oxidative capacity [12,13].

However, this association was attenuated after multivariable adjustment. This finding likely reflects the limited sample size of the pioglitazone subgroup (n = 11) and the exploratory nature of the study, rather than the absence of a biologically plausible effect. Previous studies have reported heterogeneous findings regarding the impact of thiazolidinediones on body composition, with some suggesting improvements in lean mass and others highlighting increases in fat mass and fluid retention [11,15]. A critical methodological concern with pioglitazone studies is whether observed gains in lean mass genuinely reflect muscle tissue accrual or are instead artifactual, driven by extracellular fluid accumulation—a known side effect of thiazolidinedione therapy. In the present study, however, ECF delta did not differ significantly between pioglitazone users and non-users (+0.15 kg vs. −0.13 kg; *p* = 0.095), and hematocrit changes were similarly comparable between groups (*p* = 0.580). This pattern strongly argues against fluid retention as the primary explanation for the observed SMM increase, lending greater biological plausibility to the lean tissue interpretation.

In addition, preclinical data on pathways involving insulin signaling, AMPK activation, and muscle metabolism remain complex and at times conflicting [14,15,16,17]. Taken together, our findings align with the hypothesis that pioglitazone may influence skeletal muscle physiology, although well-powered, longer-term investigations will be necessary to establish the magnitude and clinical significance of this relationship.

### 4.2. SGLT2 Inhibitors and Hemoglobin Regulation

In contrast to pioglitazone, SGLT2 inhibitor initiation was not associated with significant changes in SMM, which is consistent with previous human studies demonstrating largely neutral effects on lean mass despite reductions in body weight [18,19]. However, a significant increase in hemoglobin was observed, and this association remained robust after adjustment for key confounders. It should be noted that the observed median Hb increment was +0.10 g/dL, which is modest in absolute terms and below the threshold typically considered clinically meaningful for individual patients. Nevertheless, the statistical robustness of the association (OR 4.22; 95% CI 1.32–13.44; *p* = 0.015) and its consistency with findings from large-scale randomized trials suggest that this reflects a genuine erythropoietic signal rather than random variation. The clinical significance of such incremental Hb changes may be more relevant at a population level—particularly for patients with borderline or low baseline hemoglobin—and warrants prospective evaluation with longer follow-up. Of particular mechanistic interest, hematocrit delta did not reach statistical significance between SGLT2-i users and non-users (+1.15% vs. −0.10%; *p* = 0.088), and extracellular fluid changes were comparable across groups (*p* = 0.221). This dissociation between hemoglobin elevation and the absence of significant ECF reduction or hematocrit change challenges a purely hemoconcentration-based explanation and provides indirect, real-world evidence in favor of a genuine erythropoietic effect—consistent with emerging mechanistic data on renal hypoxia-inducible factor signaling.

This finding is in line with the well-established hematologic effects of SGLT2 inhibitors, which are thought to involve renal hypoxia-inducible factor signaling, reduced tubular oxygen consumption, and increased erythropoietin production [20,21,22]. The magnitude and direction of Hb changes observed in this study are consistent with large cardiovascular outcome and heart failure trials, where SGLT2 inhibitors have been shown to increase hemoglobin and hematocrit independent of glycemic control [23,24,25,26]. More recent real-world evidence further supports the hematologic effects of SGLT2 inhibitors. In a large single-center retrospective cohort of 6787 patients prescribed SGLT2 inhibitors, erythrocytosis developed in 16.9% of cases, with a median hemoglobin rise of 1.0 g/dL; male sex, elevated BMI, and current smoking were identified as independent risk factors [27]. Complementing these findings, a propensity score-matched cohort study of 269,064 patients with type 2 diabetes demonstrated that SGLT2 inhibitor initiation was associated with a significantly higher prevalence of erythrocytosis compared with DPP-4 inhibitors and GLP-1 receptor agonists, without a corresponding increase in thrombotic events [28].

Importantly, the present study extends these observations to a real-world cohort in a middle-income setting, where prescribing patterns, background therapies, and patient characteristics may differ from those in randomized controlled trials. Collectively, these findings reinforce the reproducibility of SGLT2 inhibitor-related hematologic effects across diverse clinical contexts and may have implications for hemoglobin monitoring in patients initiating SGLT2 inhibitor therapy [27,28].

### 4.3. Other Oral Antidiabetic Drug Classes

No meaningful changes in SMM or hemoglobin were observed among patients treated with metformin, DPP-4 inhibitors, or sulfonylureas. The weight-neutral profile of DPP-4 inhibitors observed in this study is consistent with their incretin-based mechanism of action [29,30,31]. Previous reports have also suggested modest or inconsistent effects of these agents on hematologic parameters [32,33,34]. The treatment patterns observed in this cohort reflect real-world prescribing trends in Türkiye and highlight the importance of contextualizing drug effects within routine clinical practice [35,36].

### 4.4. Strengths and Limitations

This study has several strengths. It was designed prospectively and reflects real-world prescribing behavior, enhancing its clinical relevance. Body composition was assessed using validated multi-frequency bioelectrical impedance analysis, allowing evaluation of outcomes beyond glycemic control. The study provides both a hypothesis-generating signal for a potential association between pioglitazone and SMM and a confirmatory signal for SGLT2 inhibitor-related increases in hemoglobin.

However, several limitations should be considered. The non-randomized design introduces the possibility of residual confounding despite adjustment for key variables. The relatively small sample size, particularly in the pioglitazone group (n = 11), limits statistical power and results in wide confidence intervals. Furthermore, the events-per-variable ratio in the pioglitazone logistic regression model was below the commonly recommended threshold of 10 (7 events across 5 covariates), which may have rendered the model unstable; accordingly, the adjusted OR for pioglitazone should be interpreted with particular caution. Dichotomization of continuous variables in regression analyses may have reduced sensitivity to detect subtle effects. Body composition was assessed using bioelectrical impedance analysis, which, while validated and widely used in clinical practice, is sensitive to changes in hydration status. Given the known natriuretic and diuretic effects of SGLT2 inhibitors, alterations in extracellular fluid may have influenced BIA-derived skeletal muscle mass estimates; however, the absence of significant between-group differences in ECF delta in the present study argues against a major confounding effect. Medication adherence was not formally assessed through objective measures such as pill counts, pharmacy refill records, or biological markers; treatment continuity was inferred from clinical documentation and prescription records at each visit. The six-month follow-up period may be insufficient to capture longer-term adaptations in skeletal muscle or erythropoiesis. In addition, muscle strength was not assessed, which may have provided complementary functional information. Finally, the single-center design may limit generalizability.

### 4.5. Clinical Implications and Future Directions

These findings support a physiology-oriented perspective in the management of T2DM, in which drug selection may consider not only glycemic efficacy but also potential effects on body composition and hematologic parameters. Future studies with larger sample sizes, longer follow-up durations, and multicenter designs are needed to confirm these observations and to explore their clinical implications, particularly in populations with limited access to newer therapies.

## 5. Conclusions

In this prospective real-world cohort, pioglitazone initiation was associated with a trend toward increased skeletal muscle mass in unadjusted analysis; however, this association was attenuated after multivariable adjustment and should be regarded as a hypothesis-generating signal. SGLT2 inhibitor use was associated with a statistically robust increase in hemoglobin, though the absolute magnitude of change was modest. These findings suggest that commonly used oral antidiabetic drugs may exert class-specific physiological effects beyond glycemic control. Given the non-randomized design and limited sample size, these results should be considered hypothesis-generating. Larger, controlled studies are warranted to confirm these observations and clarify their clinical relevance.

## Figures and Tables

**Table 1:** Baseline demographic and clinical characteristics of the study cohort.

Variable	Overall (n = 60)	Pioglitazone (n = 11)	Non-Pioglitazone (n = 49)	SGLT2-i (n = 28)	Non-SGLT2-i (n = 32)
Age (years), median (Q1–Q3)	53.0 (49.0–58.0)	56.0 (50.5–58.5)	53.0 (49.0–58.0)	54.0 (50.0–58.2)	52.0 (47.8–57.2)
Female sex, n (%)	34 (56.7%)	8 (72.7%)	26 (53.1%)	16 (57.1%)	18 (56.2%)
BMI (kg/m^2^), median (Q1–Q3)	30.7 (27.9–35.0)	34.7 (30.5–39.1)	30.1 (27.5–34.3)	30.0 (27.2–33.1)	32.8 (28.2–37.0)
HbA1c (%), median (Q1–Q3)	7.5 (6.6–8.4)	7.8 (7.2–8.8)	7.0 (6.6–8.3)	7.8 (6.9–9.7)	6.9 (6.5–7.8)
Hemoglobin (g/dL), median (Q1–Q3)	13.9 (12.9–14.7)	13.4 (13.2–13.9)	14.0 (12.8–14.9)	13.9 (12.7–14.9)	13.7 (13.2–14.4)
eGFR (mL/min/1.73 m^2^), median (Q1–Q3)	101.0 (91.8–109.0)	104.0 (98.5–108.5)	101.0 (91.0–109.0)	100.5 (87.8–108.2)	102.5 (92.8–110.0)
SMM (kg), median (Q1–Q3)	31.0 (27.3–34.2)	30.3 (28.0–32.6)	31.2 (27.2–34.8)	29.4 (26.5–35.1)	31.6 (28.1–33.1)
Prior OAD use, n (%)	29 (48.3%)	8 (72.7%)	21 (42.9%)	18 (64.3%)	11 (34.4%)
Duration of prior OAD use (years), median (range) ^a^	3 (1–10)	4 (2–10)	2 (1–10)	2 (1–10)	4 (1–10)
CAD/MI, n (%)	22 (36.7%)	5 (45.5%)	17 (34.7%)	13 (46.4%)	9 (28.1%)
Diabetic nephropathy, n (%)	17 (28.3%)	1 (9.1%)	16 (32.7%)	10 (35.7%)	7 (21.9%)
Diabetic neuropathy, n (%)	32 (53.3%)	8 (72.7%)	24 (49.0%)	16 (57.1%)	16 (50.0%)
Diabetic retinopathy, n (%)	9 (15.0%)	0 (0.0%)	9 (18.4%)	5 (17.9%)	4 (12.5%)

Continuous variables are presented as median (Q1–Q3). Categorical variables are presented as n (%). ^a^ Among patients with prior OAD use only. BMI: body mass index; CAD: coronary artery disease; eGFR: estimated glomerular filtration rate; MI: myocardial infarction; OAD: oral antidiabetic drug; SMM: skeletal muscle mass.

**Table 2:** Comparison of key parameters for all patients at baseline and 6 months.

Variable	Baseline	6th Month	*p* Value
Weight (kg)	83.46 ± 12.72	81.68 ± 12.76	0.012
BMI (kg/m^2^)	32.03 ± 6.33	31.46 ± 6.02	0.006
Hb (g/dL)	13.80 ± 1.68	13.78 ± 1.59	0.659
HbA1c (%)	7.68 ± 1.56	6.70 ± 1.08	<0.001
Fasting blood glucose (mg/dL)	150.95 ± 43.76	132.47 ± 36.55	0.007
Albumin (g/L)	4.51 ± 0.23	4.64 ± 0.24	<0.001
Skeletal muscle mass (kg)	31.22 ± 4.82	30.90 ± 4.71	0.029
Extracellular fluid (kg)	17.05 ± 2.61	16.90 ± 2.57	0.055

Values are mean ± SD. Hb: hemoglobin; BMI: body mass index.

**Table 3:** Comparison of key outcomes in patients started on pioglitazone.

Variable	No Pioglitazone (N = 49) Median (IQR)	Newly Started Pioglitazone (N = 11) Median (IQR)	*p* Value
Hemoglobin delta (g/dL)	−0.10 (−0.60 to +0.40)	−0.30 (−1.05 to +0.10)	0.482
Skeletal muscle mass delta (kg)	−0.36 (−1.04 to +0.18)	+0.17 (−0.55 to +0.50)	0.050
HbA1c delta (%)	−1.0 (−2.1 to −0.2)	−1.2 (−2.3 to −0.4)	0.744
HCT delta (%)	+0.40 (−1.30 to +1.70)	−0.30 (−0.85 to +0.65)	0.580
ECF delta (kg)	−0.13 (−0.60 to +0.19)	+0.15 (−0.31 to +0.35)	0.095

Values represent delta (change from baseline to 6 months; follow-up minus baseline). Values in parentheses are interquartile ranges. *p* values were obtained using the Mann–Whitney U test. Statistically significant *p* values are shown in bold. ECF: extracellular fluid; HCT: hematocrit; IQR: interquartile range.

**Table 4:** Comparison of key outcomes in patients started on SGLT2 inhibitors.

Variable	Not Using SGLT2-i (N = 32) Median (IQR)	Newly Started SGLT2-i (N = 28) Median (IQR)	*p* Value
Hemoglobin delta (g/dL)	−0.10 (−0.60 to +0.30)	+0.10 (−0.30 to +0.50)	**0.022**
Skeletal muscle mass delta (kg)	−0.33 (−1.05 to +0.30)	−0.07 (−0.92 to +0.25)	0.386
HbA1c delta (%)	−1.2 (−2.3 to −0.4)	−1.0 (−2.0 to −0.1)	0.553
HCT delta (%)	−0.10 (−1.40 to +0.82)	+1.15 (−0.53 to +2.10)	0.088
ECF delta (kg)	−0.07 (−0.36 to +0.27)	−0.05 (−0.71 to +0.15)	0.221

Values represent delta (change from baseline to 6 months; follow-up minus baseline). Values in parentheses are interquartile ranges. *p* values were obtained using the Mann–Whitney U test. Statistically significant *p* values are shown in bold. ECF: extracellular fluid; HCT: hematocrit; SGLT2-i: sodium–glucose cotransporter-2 inhibitor; IQR: interquartile range.

## Data Availability

The datasets generated and analyzed during the current study are available from the corresponding author upon reasonable request (pinarziyadanoglu@hotmail.com). Sharing of individual-level patient data is subject to ethical approval from the Haseki Training and Research Hospital Ethics Committee and requires a signed data sharing agreement to protect patient confidentiality, in accordance with Turkish personal data protection legislation (KVKK). Requests will be evaluated on a case-by-case basis and data will be provided in anonymized form where feasible.

## References

[1] Saeedi P., Petersohn I., Salpea P., Karuranga S., Malanda B., Gregg E.W., Bright D., Williams R., IDF Diabetes Atlas Committee (2019). Global and regional diabetes prevalence estimates for 2019 and projections for 2030 and 2045: Results from the International Diabetes Federation Diabetes Atlas, 9th edition. Diabetes Res. Clin. Pract..

[2] American Diabetes Association (2014). Diagnosis and classification of diabetes mellitus. Diabetes Care.

[3] Solis-Herrera C., Alvarez-Perez J.C., Munoz A., Abdul-Ghani M., DeFronzo R.A., Feingold K.R., Anawalt B., Boyce A. (2021). Pathogenesis of type 2 diabetes mellitus. Endotext.

[4] Blaslov K., Naranda F.S., Kruljac I., Pavlovic T. (2018). Treatment approach to type 2 diabetes: Past, present and future. World J. Diabetes.

[5] Shaw J.E., Sicree R.A., Zimmet P.Z. (2010). Global estimates of the prevalence of diabetes for 2010 and 2030. Diabetes Res. Clin. Pract..

[6] Ai Y., Xu R., Liu L. (2021). The prevalence and risk factors of sarcopenia in patients with type 2 diabetes mellitus: A systematic review and meta-analysis. Diabetol. Metab. Syndr..

[7] Anagnostis P., Gkekas N.K., Achilla C., Papanastasiou G., Taouxidou P., Mitsiou M., Kenanidis E., Potoupnis M., Tsiridis E., Goulis D.G. (2020). Type 2 diabetes mellitus is associated with increased risk of sarcopenia: A systematic review and meta-analysis. Calcif. Tissue Int..

[8] Türkiye Endokrinoloji ve Metabolizma Derneği (2022). Diabetes Mellitus ve Komplikasyonlarının Tanı Tedavi ve İzlem Kılavuzu 2022.

[9] De Rivas B., Luque M., Martell N., Fernández C., Fernández-Cruz A., García L., Rodicio J.L. (2007). Pioglitazone decreases ambulatory blood pressure in type 2 diabetics with difficult-to-control hypertension. J. Clin. Hypertens..

[10] Singaram V., Pratley R.E. (2007). The PROactive trial: What does it mean for primary care physicians?. Diabetes Vasc. Dis. Res..

[11] DeFronzo R.A., Inzucchi S.E., Abdul-Ghani M., Nissen S.E. (2019). Pioglitazone: The forgotten, cost-effective cardioprotective drug for type 2 diabetes. Diabetes Vasc. Dis. Res..

[12] Yokota T., Kinugawa S., Hirabayashi K., Suga T., Takada S., Omokawa M., Kadoguchi T., Takahashi M., Fukushima A., Matsushima S. (2017). Pioglitazone improves whole-body aerobic capacity and skeletal muscle energy metabolism in patients with metabolic syndrome. J. Diabetes Investig..

[13] Fiorentino T.V., Monroy A., Kamath S., Sotero R., Cas M.D., Daniele G., Chavez A.O., Abdul-Ghani M., Hribal M.L., Sesti G. (2021). Pioglitazone corrects dysregulation of skeletal muscle mitochondrial proteins involved in ATP synthesis in type 2 diabetes. Metabolism.

[14] Alghanem L., Zhang X., Jaiswal R., Seyoum B., Mallisho A., Msallaty Z., Yi Z. (2022). Effect of insulin and pioglitazone on protein phosphatase 2A interaction partners in primary human skeletal muscle cells derived from obese insulin-resistant participants. ACS Omega.

[15] Zanchi A., Maillard M., Jornayvaz F.R., Nussberger J., Brunner H.R., Burnier M., Pechere-Bertschi A. (2010). Effects of the peroxisome proliferator-activated receptor (PPAR)-gamma agonist pioglitazone on renal and hormonal responses to salt in diabetic and hypertensive individuals. Diabetologia.

[16] Marková I., Zídek V., Musilová A., Šimáková M., Mlejnek P., Kazdová L., Pravenec M. (2010). Long-term pioglitazone treatment augments insulin sensitivity and PKC-ε and PKC-θ activation in skeletal muscles in sucrose-fed rats. Physiol. Res..

[17] Tan L., Song A., Ren L., Wang C., Song G. (2020). Effect of pioglitazone on skeletal muscle lipid deposition in the insulin resistance rat model induced by high fructose diet under AMPK signaling pathway. Saudi J. Biol. Sci..

[18] Schork A., Saynisch J., Vosseler A., Jaghutriz B.A., Heyne N., Peter A., Häring H.-U., Stefan N., Fritsche A., Artunc F. (2019). Effect of SGLT2 inhibitors on body composition, fluid status, and renin-angiotensin-aldosterone system in type 2 diabetes: A prospective study using bioimpedance spectroscopy. Cardiovasc. Diabetol..

[19] Volpe S., Vozza A., Lisco G., Fanelli M., Racaniello D., Bergamasco A., Triggiani D., Pierangeli G., De Pergola G., Tortorella C. (2024). Sodium-glucose cotransporter 2 inhibitors improve body composition by increasing the skeletal muscle mass/fat mass ratio in patients with type 2 diabetes: A 52-week prospective real-life study. Nutrients.

[20] Ekanayake P., Mudaliar S. (2023). Increase in hematocrit with SGLT-2 inhibitors—Hemoconcentration from diuresis or increased erythropoiesis after amelioration of hypoxia?. Diabetes Metab. Syndr. Clin. Res. Rev..

[21] Fonseca-Correa J.I., Correa-Rotter R. (2021). Sodium-glucose cotransporter 2 inhibitors mechanisms of action: A review. Front. Med..

[22] Stefánsson B.V., Heerspink H.J., Wheeler D.C., Sjöström C.D., Greasley P.J., Sartipy P., Cain V., Correa-Rotter R. (2020). Correction of anemia by dapagliflozin in patients with type 2 diabetes. J. Diabetes Complicat..

[23] Ghanim H., Abuaysheh S., Hejna J., Green K., Makdissi A., Chaudhuri A., Dandona P. (2020). Dapagliflozin suppresses hepcidin and increases erythropoiesis. J. Clin. Endocrinol. Metab..

[24] Packer M., Anker S.D., Butler J., Filippatos G., Zannad F. (2022). Alleviation of functional iron deficiency by SGLT2 inhibition in patients with type 2 diabetes. Diabetes Obes. Metab..

[25] Docherty K.F., Curtain J.P., Anand I.S., Bengtsson O., Inzucchi S.E., Køber L., Kosiborod M.N., Langkilde A.M., Martinez F.A., Ponikowski P. (2021). Effect of dapagliflozin on anemia in DAPA-HF. Eur. J. Heart Fail..

[26] Maruyama T., Takashima H., Oguma H., Nakamura Y., Ohno M., Utsunomiya K., Furukawa T., Tei R., Abe M. (2019). Canagliflozin improves erythropoiesis in diabetes patients with anemia of chronic kidney disease. Diabetes Technol. Ther..

[27] Lee J.Y., Lee J.-H., Jung E.-J., Park W., Seo J., Kang M., Jung E.H., Kim S.-A., Suh K.J., Kim J.-W. (2025). Prevalence and thrombotic risk of SGLT-2 inhibitor-associated erythrocytosis: A retrospective cohort study. Cardiovasc. Diabetol..

[28] Lewis M., Burrack N., Heymann A., Grossman A., Neuman T., Abuhasira R. (2025). Sodium-glucose cotransporter 2 inhibitors, erythrocytosis, and thrombosis in adults with type 2 diabetes. JAMA Netw. Open.

[29] De Jong M., Van Der Worp H.B., Van Der Graaf Y., Visseren F.L., Westerink J. (2017). Pioglitazone and the secondary prevention of cardiovascular disease: A meta-analysis of randomized controlled trials. Cardiovasc. Diabetol..

[30] Haddad F., Dokmak G., Bader M., Karaman R. (2023). A comprehensive review on weight loss associated with anti-diabetic medications. Life.

[31] Gilbert M.P., Pratley R.E. (2020). GLP-1 analogs and DPP-4 inhibitors in type 2 diabetes therapy: Review of head-to-head clinical trials. Front. Endocrinol..

[32] Zeng L.M., Chan G.C.M., Ng J.K.M., Fung W.W.M., Chow K.-M.M., Szeto C.-C.M. (2023). The effect of dipeptidyl peptidase 4 (DPP-4) inhibitors on hemoglobin level in diabetic kidney disease: A retrospective cohort study. Medicine.

[33] Jones B., Adams S., Miller G.T., Jesson M.I., Watanabe T., Wallner B.P. (2003). Hematopoietic stimulation by a dipeptidyl peptidase inhibitor reveals a novel regulatory mechanism and therapeutic treatment for blood cell deficiencies. Blood.

[34] Merdin F.A., Merdin A. (2023). Do DPP-4 enzyme inhibitors affect hemoglobin, leucocyte, and thrombocyte levels in patients with type 2 diabetes mellitus?. Eur. Rev. Med. Pharmacol. Sci..

[35] Shubrook J.H., Radin M., Ali S.N., Hasford J., Johansen P., Pfeiffer A.F. (2022). Preference for type 2 diabetes therapies in the United States: A discrete choice experiment. Adv. Ther..

[36] Haymana C., Sonmez A., Demirci I., Yaylalı G.F., Nuhoglu I., Sancak S., Yilmaz M., Altuntas Y., Dinccag N., Sabuncu T. (2021). Patterns and preferences of antidiabetic drug use in Turkish patients with type 2 diabetes: A nationwide cross-sectional study (TEMD treatment study). Diabetes Res. Clin. Pract..

